# Avaliação Ecocardiográfica Transesofágica Bi e Tridimensional de Dissecção Espontânea do Átrio Esquerdo

**DOI:** 10.36660/abc.20210740

**Published:** 2022-08-25

**Authors:** Javier Ivan Armenta-Moreno, Joaquin Berarducci, Abel Mauricio Garcia-Cardenas, José Carlos Armendariz-Ferrari, Jorge Luis Bermudez-Gonzalez, Juan Ignacio Straface, Jose Antonio Luna-Alvarez-Amezquita, Nilda Espinola-Zavaleta

**Affiliations:** 1 Departamento de Cardiologia Nuclear Instituto Nacional de Cardiologia Ignacio Chavez Cidade do México México Departamento de Cardiologia Nuclear – Instituto Nacional de Cardiologia Ignacio Chavez, Cidade do México – México; 2 Departamento de Cardiologia Clínica e Ecocardiografia Hospital Nacional Hipólito Unanue Lima Peru Departamento de Cardiologia Clínica e Ecocardiografia – Hospital Nacional Hipólito Unanue, Lima – Peru; 3 Departamento de Ecocardiografia Centro Médico ABC I.A.P. Cidade do México México Departamento de Ecocardiografia – Centro Médico ABC I.A.P., Cidade do México – México

**Keywords:** Insuficiência Cardíaca/fisiopatologia, Função do Átrio Esquerdo/fisiologia, Diagnóstico por Imagem/métodos, Ecocardiografia Tridimensional/métodos, Choque Cardiogênico

Uma mulher de 41 anos de idade veio ao atendimento de emergência com início de dispneia aguda, ingurgitamento jugular, e com o primeiro som cardíaco diminuído, seguido de um sopro holossistólico de grau III/IV que era melhor ouvido no vértice e no edema das extremidades inferiores. Os sinais vitais eram frequência cardíaca de 91 bpm, frequência respiratória de 21 rpm, pressão arterial 110/60 mmHg e saturação de oxigênio de 91%. O raio X do tórax mostrou cardiomegalia com um índice cardiotorácico de 0,62, e hipertensão venocapilar pulmonar.

A ecocardiografia transtorácica bidimensional mostrou uma lesão com aparência de cisto, ocupando um espaço com parede fina no átrio esquerdo ( Figura 1 , Painéis A e B), regurgitação mitral moderada, função sistólica ventricular de 68% e efusão pleural esquerda ( Figura 1 , Painéis C e D). Uma técnica transesofágica (TEE) a 60° ( Figura 1 , Painel E) e a 90% ( Figura 1 , Painel F) foi realizada para se obter uma melhor caracterização que confirmou a presença de uma massa com aparência de cisto não-homogêneo no átrio esquerdo envolvendo o anel mitral posterior e ocupando aproximadamente 60% da câmara atrial (Vídeo 1). O Doppler colorido revelou fluxo sanguíneo na direção dessa pseudocavidade ( Figura 1 , Painel G). A TEE 3D vista do cirurgião mostrou claramente uma pseudocavidade dentro do átrio esquerdo que aparecia e desaparecia em relação ao ciclo cardíaco ( Figura 2 , Painel A) e incluía o segmento póstero-medial do anel mitral ( Figura 2 , Painel B) (Vídeo 2).

**Figura 1:**
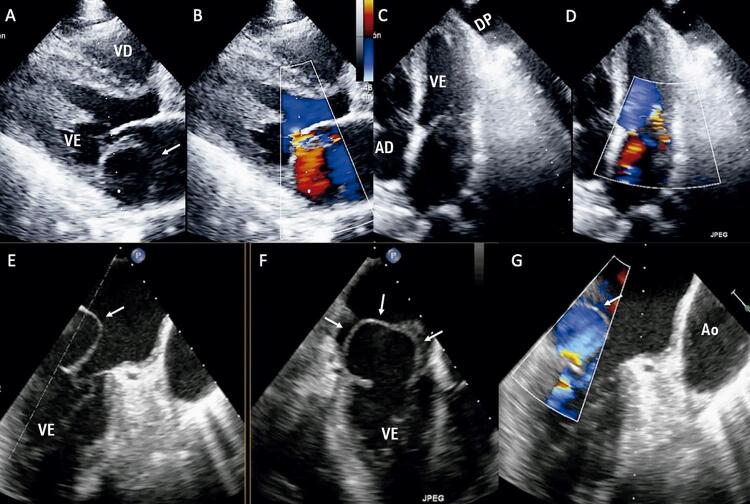
Ecocardiograma com Doppler 2D transtorácico no corte paraesternal eixo longo mostrando átrio de paredes finas tipo cisto (A) e regurgitação mitral moderada (B). Na 4 câmara apical foi visualizado derrame pleural esquerdo (C) e também regurgitação mitral moderada (D). Imagens transesofágicas 2D a 60º e 90º (E, F) confirmaram a presença de uma massa heterogênea tipo cisto (setas brancas) no átrio esquerdo envolvendo o anel mitral posterior. O Doppler colorido revelou fluxo sanguíneo para esta pseudocavidade (G). VE: ventrículo esquerdo; AE: átrio esquerdo; AD: átrio direito; VD: ventrículo direito; Ao: aorta; DP: derrame pleural.

**Figura 2:**
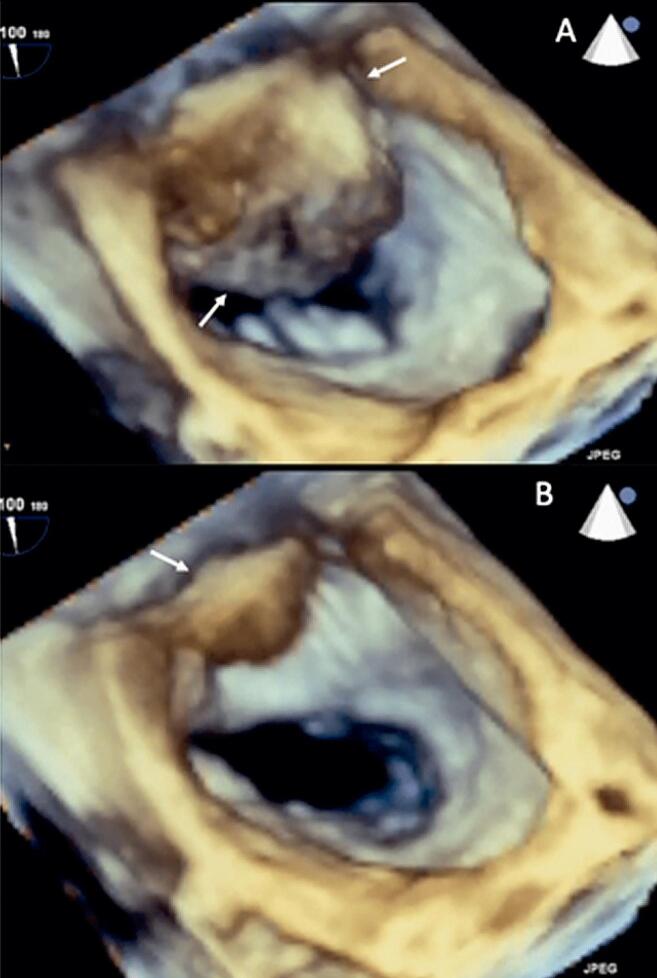
Ecocardiografia 3D transesofágica na visão do cirurgião com pseudocavidade dentro do átrio esquerdo aparecendo e desaparecendo (setas brancas) em relação ao ciclo cardíaco (A) e compreendendo o segmento póstero-medial do anel mitral (setas brancas), (B).

O tratamento médico para insuficiência cardíaca aguda foi iniciado com resposta fraca e evolução para choque cardiogênico. A paciente foi levada imediatamente para o bloco cirúrgico.

Foi executada uma abordagem de emergência de acordo com os achados ecocardiográficos. Foi realizada uma pericardiotomia, foi iniciada uma abordagem do átrio esquerdo, em que o se observou perfuração de P2 e P3 no folheto retraído e fibrótico anterior, com evidência do orifício de dissecção no anel e na parede posterior do átrio.

A gestão do controle de danos e o sistema de suporte à vida foram iniciados com resposta fraca e, infelizmente, a paciente morreu durante a cirurgia.

A dissecção espontânea do átrio esquerdo é uma doença extremamente rara e deve ser suspeitada como causa incomum da insuficiência cardíaca aguda. Sua incidência real, fisiopatologia, evolução clínica e gestão não são entendidas satisfatoriamente.^1 , 2^ A TEE, especialmente o método 3D, é a modalidade diagnóstica de escolha para essa entidade. Antes da era da TEE, o diagnóstico era baseado em achados intraoperatórios brutos ou achados autopsiais incidentais.^2 , 3^

## * Material suplementar

Para assistir ao vídeo suplementar 1, por favor, clique aqui .

Para assistir ao vídeo suplementar 2, por favor, clique aqui .
