# Supplementary material for: Avaliação Ecocardiográfica Transesofágica Bi e Tridimensional de Dissecção Espontânea do Átrio Esquerdo
Source: Arq Bras Cardiol. 2022 Aug 25;119(3):499–501. [Article in Portuguese] doi: 10.36660/abc.20210740 (PMC9438539; doi:10.36660/abc.20210740)
Supplement: Translation

# Translation: Transesophageal Two- and Three-Dimensional Echocardiographic Assessment of Spontaneous Left Atrial Dissection

**Authors:** Javier Ivan Armenta-Moreno, Joaquin Berarducci, Abel Mauricio Garcia-Cardenas, José Carlos Armendariz-Ferrari, Jorge Luis Bermudez-Gonzalez, Juan Ignacio Straface, Jose Antonio Luna-Alvarez-Amezquita, Nilda Espinola-Zavaleta

**Affiliations:** 1 Mexico Departamento de Cardiologia Nuclear – Instituto Nacional de Cardiologia Ignacio Chavez, Ciudad de México – Mexico; 2 Peru Departamento de Cardiologia Clínica y Ecocardiografia – Hospital Nacional Hipólito Unanue, Lima – Peru; 3 Mexico Departamento de Ecocardiografia – Centro Médico ABC I.A.P., Ciudad de México – Mexico

**Keywords:** Heart Failure/physiopathology, Atrial Function, Left/physiology, Diagnostic, Imaging/methods, Echocardiography Three-Dimensional/methods, Shock, Cardiogenic

A 41-year-old woman was admitted to the emergency room with an onset of acute dyspnea, jugular ingurgitation, and with the first heart sound diminished, followed by a grade III/IV holosystolic murmur that was better heard at the apex and edema of the lower limbs.

The vital signs were heart rate 91 bpm, respiratory rate 21 rpm, blood pressure 110/60 mmHg, and oxygen saturation 91%. The chest x-ray showed cardiomegaly with a cardiothoracic index of 0.62 and pulmonary venocapillary hypertension.

Transthoracic 2D echocardiography showed a cyst-like, lesion occupying a thin-walled space in the left atrium ( Figure 1 , Panels A, B), moderate mitral regurgitation, ventricular systolic function of 68%, and left pleural effusion ( Figure 1 , Panels C, D). A transoesophageal technique (TEE) at 60° ( Figure 1 , Panel E) and 90° ( Figure 1 , Panel F) was performed for better characterization, which confirmed the presence of heterogeneous cyst-like mass of the left atrium, involving the posterior mitral and occupying approximately 60% of the left atrial chamber (Video 1). Color Doppler revealed a blood flow to this pseudo-cavity ( Figure 1 , Panel G). The 3D TEE, from the surgeon’s view, clearly showed a pseudo-cavity inside the left atrium appearing and disappearing in relation to the cardiac cycle ( Figure 2 , Panel A) and comprising the postero-medial segment of the mitral annulus ( Figure 2 , Panel B), (Video 2).

**Figure 1:** Transthoracic 2D Doppler echocardiography in the parasternal long axis view showing a cyst-like thin-walled atrium (A) and moderate mitral regurgitation (B). In the apical chamber a left pleural effusion (C) and also a moderate mitral regurgitation (D) were visualized. Transoesophageal 2D images at 60º and 90º (E-F) confirmed the presence of a heterogeneous cyst-like mass (white arrows) in the left atrium involving the posterior mitral annulus. The color Doppler revealed a blood flow to this cavity (G). LV: left ventricle; LA: left atrium; RA: right atrium; RV: right ventricle; Ao: aorta; PE: pleural effusion.

**Figure 2:** Transoesophageal 3D echocardiography in the surgeon view with a pseudo-cavity inside the left atrium appearing and disappearing (white arrows) in relation to the cardiac cycle (A) and comprising the postero-medial segment of the mitral annulus (white arrows), (B).

Medical treatment for acute heart failure was initiated with poor response and progression to cardiogenic shock. The patient was taken immediately to the operating room.

An emergency approach was executed according to the echocardiographic findings. A pericardiotomy was performed, an approach to the left atrium was initiated, in which the posterior fibrotic and retracted leaflet was observed with a perforation in P2 and P3 and evidence of a dissection hole in the annulus and posterior wall of the atrium.

Management of damage control and life support was initiated with poor response and, unfortunately, the patient died during surgery.

Spontaneous left atrial dissection is an extremely rare disease and must be suspected as an uncommon cause of acute heart failure. Its true incidence, pathophysiology, clinical course, and management is poorly understood.^1 , 2^ TEE, especially the 3D method, is the diagnostic modality of choice for this entity, before the era of TEE, the diagnosis was based on gross intraoperative findings or incidental autopsy findings.^2 , 3^

## * Supplemental Materials

See the Supplemental Video 1, please click here .

See the Supplemental Video 2, please click here .
